# Latent Order in High-Angle Grain Boundary of GaN

**DOI:** 10.1038/s41598-018-22603-3

**Published:** 2018-03-15

**Authors:** Sangmoon Yoon, Hyobin Yoo, Seoung-Hun Kang, Miyoung Kim, Young-Kyun Kwon

**Affiliations:** 1 Department of Materials Science and Engineering, Seoul National University, Seoul, 08826 Korea; 2 Department of Physics and Research Institute for Basic Sciences, Kyung Hee University, Seoul, 02447 Korea; 3 Korea Institute for Advanced Study (KIAS), Seoul, 02455 Korea

## Abstract

We report the existence of latent order during core relaxation in the high-angle grain boundaries (GBs) of GaN films using atomic-resolution scanning transmission electron microscopy and *ab initio* density functional theory calculations. Core structures in the high-angle GBs are characterized by two pairs of Ga-N bonds located next to each other. The core type correlates strongly with the bond angle differences. We identify an order of core relaxation hidden in the high-angle GBs by further classifying the 5/7 atom cores into a stable 5/7 core (5/7(S)) and a metastable 5/7 core (5/7(M)). This core-type classification indicates that metastable cores can exist at real high-angle GBs under certain circumstances. Interestingly, 5/7(M) exhibits distinct defect states compared to 5/7(S), despite their similar atomic configurations. We investigate the reconstruction of defect states observed in 5/7(M) by analyzing the real-space wave functions. An inversion occurred between two localized states during the transition from 5/7(S) to 5/7(M). We suggest an inversion mechanism to explain the formation of new defect states in 5/7(M).

## Introduction

GaN films have been widely used in commercial devices, such as blue light-emitting diodes (LEDs)^1,2^ and high-electron-mobility transistors^3^, due to their remarkable physical properties including a direct wide band gap, high saturation velocity, and high two-dimensional electron gas densities. To advance new applications, efforts have focused on growing GaN films on unconventional substrate, such as glass substrates for large-scale fabrication^4–6^, diamond substrates for high-power applications^7–10^, and van der Waals layered substrates for transferable or flexible devices^11–15^. GaN films grown on an amorphous or polycrystalline substrate have high-angle tilt grain boundaries (GBs) due to the formation of randomly oriented islands during the nucleation of GaN films^4–10,13–15^. Applications in advanced electronic and optoelectronic devices require knowledge on the high-angle GB, for which accurate determination of the atomic configuration of the core structure is essential.

GaN films exhibit excellent luminescence, despite the presence of high-density threading edge dislocations (EDs)^16,17^, thus, extensive research has focused on isolated EDs. Studies based on high-resolution transmission electron microscopy (HRTEM) analysis indicated that isolated EDs have two types of core structure: 5/7 and 8 atom cores^18–21^, whereas EDs obtained using high-angle annular dark field (HAADF) scanning transmission electron microscopy (STEM) without post-processing^22,23^ consist of only 8 atom cores. Moreover, several theoretical studies using density functional theory (DFT) have also suggested that 8 atom cores, with shallow states near the valence band maximum, represent the most stable structure at the EDs^24–29^. There is a consensus that stoichiometric threading EDs are not active; instead, vacancies accumulated in the cores cause broad yellow luminescence^30,31^. In contrast, most empirical potential calculations suggest that the 5/7 atom core is more stable than the 8 atom core^27,32–34^. A recent study using an aberration-corrected HAADF STEM showed that EDs are composed of 5/7 atom cores only^35^, suggesting the possibility of incorrect identification in the previous studies. Thus, further research into GaN films is needed to understand how the atomic configurations at EDs change under different growth conditions and on various substrates.

In comparison with EDs, only a few studies have focused on GaN GBs because the atomic configurations of cores in the GBs were thought to be identical to those in EDs. The atomic configurations of high-angle GBs and isolated EDs were examined using HRTEM^18,21^. It was observed that the 5/7 and 8 atom cores existed in both isolated EDs and high-angle GBs, and the core structures of the two defects were indistinguishable. Additionally, Bere *et al*. calculated the formation energies of high-angle GBs and isolated EDs using an empirical potential method^36,37^. They found that the stable core structures of both defects were indistinguishable, and the 5/7 atom core was always more stable that the 8 atom core. These studies all argued the equivalence of high-angle GBs and isolated EDs. However, there is no direct evidence that the two defects have the identical core structures, because high-angle GBs have never been investigated based on HAADF STEM. There has been growing interest in advanced GaN devices containing high-angle GBs, therefore, the direct observation of their core structures is highly required.

In the previous paper, we investigated the atomic configurations of GB core structures and their corresponding electronic states using HAADF STEM and DFT calculations^38^. Experimental observations showed that the 5/7 atom core structures are regularly arranged at coincident-site-lattice (CSL) boundaries, such as Σ = 7 or Σ = 19 boundaries. Other types of core structures also frequently occur in nonperiodic GBs. Furthermore, it showed that the atomic configurations of core of high-angle tilt GBs are not identical to those of isolated EDs in contrast to the conventional knowledge^18,21,36,37^. In this paper, we report the existence of latent order for core relaxation in the GBs, which is validated by DFT calculations performed on the Σ = 7 boundary. In addition, the 5/7 atom core was classified into two types of core depending on the length of the wrong bonds. We discuss the origin of different defect states existing in two types of 5/7 atom core structures based on analysis of the real-space wave functions.

A polycrystalline GaN film grown on chemically vapor deposited (CVD) graphene using ZnO nanowalls and a low-temperature GaN buffer layer was employed for this study. The GaN film was grown at temperature of 1130 °C with hydrogen as an ambient gas. A detailed explanation of the growth process was given elsewhere^14,15,38^. A plane-view TEM sample was prepared by thinning the GaN film through mechanical polishing and additional ion milling. The atomic configurations of the high-angle tilt GBs were probed using aberration-corrected STEM operating at 200 kV. The probe convergence angle and inner collection semi-angle were 22 mrad and 90 mrad, respectively. Noises arising in HAADF STEM images were reduced by band pass filtering.

**Figure 1:**
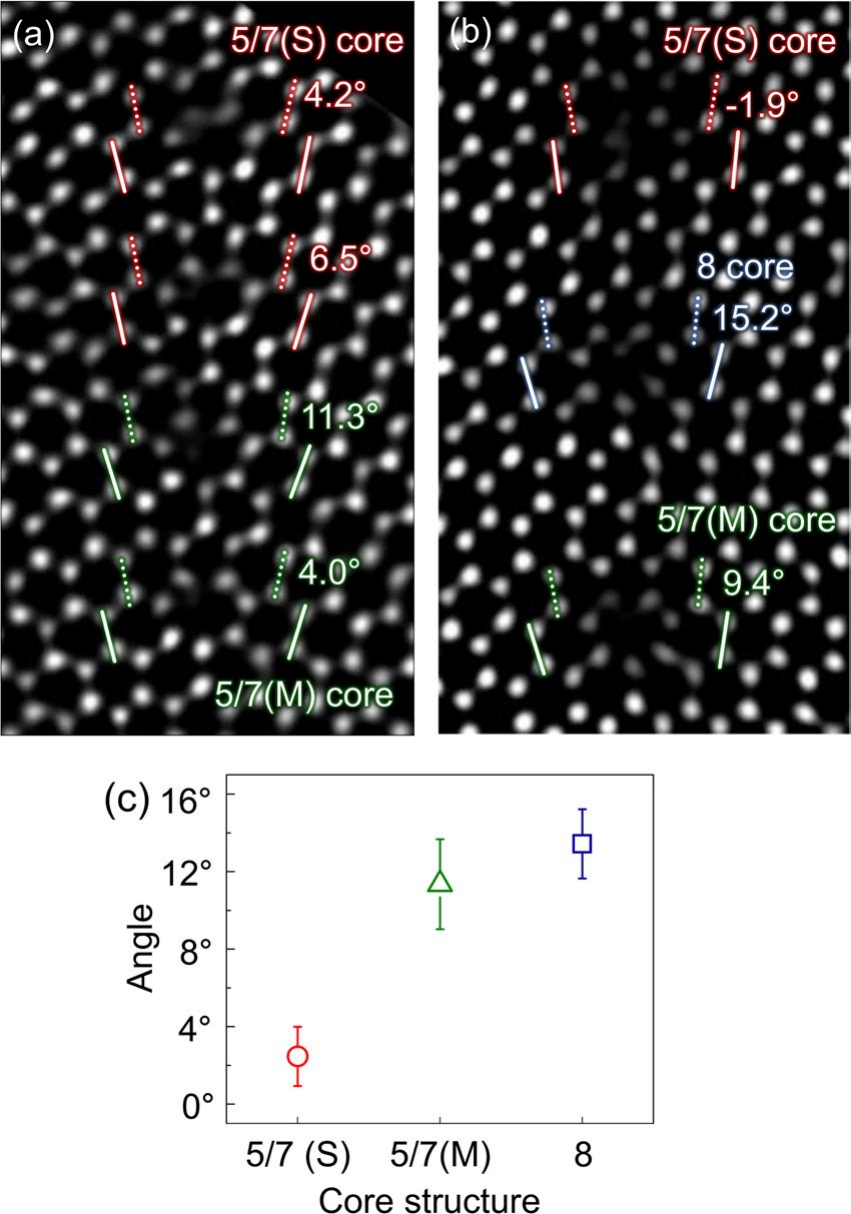
High-resolution high-angle annular dark field (HAADF) scanning transmission electron microscopy (STEM) images of the high-angle grain boundaries (GBs) in the GaN films: (**a**) Σ = 7 boundaries and (**b**) nonperiodic boundaries. Each set of solid or dashed lines denotes a bond pair used for measuring the relative angle. (**c**) Mean value and standard error for all angles measured between two bond pairs classified into each type of core structure. The colored lines in (a,b), and symbols in (c), represent the type of core: red = 5/7(S), green = 5/7(M), and blue = 8 cores.

Figure 1 shows HAADF STEM images of high-angle GBs in the GaN films and additional information on their atomic structures. There is a distinguishable latent order in each core structure of the high-angle GBs, which is determined using the two pairs of Ga-N bonds located next to each core. The angle difference between the two Ga-N bond pairs is directly correlated with the core type, which is commonly observed in both CSL and nonperiodic boundaries, as shown in Fig. 1(a,b). We further classified the 5/7 atom cores into two types depending on the length of the wrong bonds. We designated two types of 5/7 atom cores as stable and metastable. The 5/7 atom core with short wrong bonds is more stable than the one with long wrong bonds, while the latter is still thermodynamically metastable in both isolated EDs and the Σ = 7 CSL boundary. These two atom cores were denoted as 5/7(S) and 5/7(M) for the stable and metastable cores, respectively. The average angles measured in the 5/7(S), 5/7(M), and 8 atom cores are 2.5°, 11.4°, and 13.4°, respectively, and are shown in Fig. 1(c). These three types of core can be identified based on the differences in the measured angles. Our observations suggest that there is a short-range order during core relaxations in the high-angle GBs. Such core-type classification indicates that even less-stable core structures exist in real GBs if the strain concentrated on the boundaries relaxes sufficiently via rearrangement of neighboring bonds.

The 5/7(S) and 5/7(M) structures were distinguished based on the length of the wrong bond in the 5/7 atom core. The average wrong-bond lengths of 5/7(S) and 5/7(M) are calculated to be about 2.2 Å and 2.8 Å, respectively. Our *ab initio* DFT calculations show that these two relaxed 5/7 core structures occupy different defect states, as discussed in the following paragraphs. Studies have investigated these two distinctive 5/7 core structures separately, and the conclusions were incongruent^27–30,39^.

We systematically studied the correlation of the core structures with the relative angles between two neighboring bonds at the GBs using first-principles calculations within DFT^40^ as implemented in the Vienna *ab initio* simulation package (VASP)^41,42^. We successfully applied this technique to describe the growth of various surfaces of GaN in previous work^43,44^. The Σ = 7 boundary was chosen as a model GB, because it has the shortest periodicity among the CSL boundaries in the GaN films. A plane wave basis set with a cutoff energy of 500 eV was used to expand the electronic wave functions, and the valence electrons were described using the projector-augmented wave potentials^45,46^. The local density approximation (LDA) was used for the exchange-correlation functional^47^. The Γ-centered 4 × 2 × 3 Monkhorst-Pack *k*-point grid was used during geometry relaxation, and a 6 × 2 × 4 refined grid was used for the electronic properties^48^. Core structures at the Σ = 7 GB were relaxed until the Hellmann-Feynman forces acting on any atom were less than 0.02 eV/Å.

**Figure 2:**
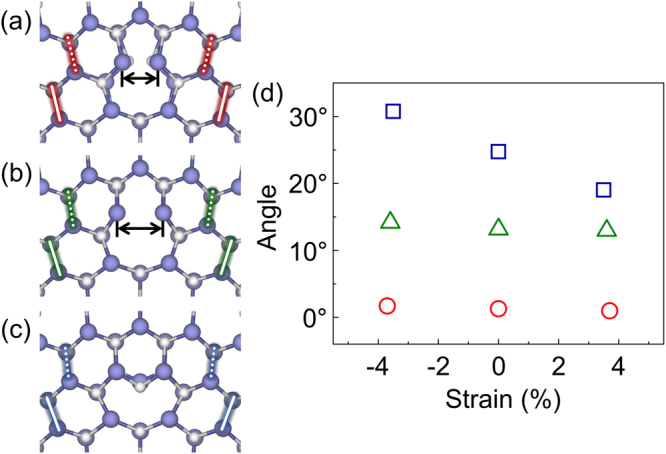
Equilibrium structures of the (**a**) 5/7(S), (**b**) 5/7(M), and (**c**) 8 atom cores in the model Σ = 7 boundary. In each structure, two pairs of solid and dotted lines denote the pairs of bonds used to measure the relative angles. The angle of each core structure in both the equilibrium and strained states (**d**). The wrong bonds in both 5/7 atom cores are marked with double-headed arrows in (a,b). The colored lines in (a)–(c), and the colored symbols in (d), denote the core type: red = 5/7(S), green = 5/7(M), and blue = 8 atom cores.

The analysis of the HAADF STEM images shown in Fig. 1 was replicated in our computational study. Figure 2(a)–(c) show the equilibrium structures of the 5/7(S), 5/7(M), and 8 atom cores of the GBs obtained by DFT calculations. In these atomic configurations, the angles between two bond sets in the three atom cores are estimated to be 1.3°, 13.2°, and 24.8°, respectively, corresponding to the data at zero % strain shown in Fig. 2(d). The calculated angles are in good agreement with those obtained by experimental observation. Moreover, the correlation between the core structures and the bond angles still indicates a tensile/compressive strain of up to 5% on the Σ = 7 GB, as shown in Fig. 2(d). This suggests the presence of a robust order for core relaxation in the high-angle GBs of GaN.

We further analyzed our calculation results to understand the latent order of core relaxation. The formation energies of the equilibrium core structures are given in Table 1. The 5/7(S) atom core has the lowest formation energy for the Σ = 7 boundary, which implies that 5/7(S) is preferentially relaxed at the GB. On the contrary, the 8 atom core is the most stable structure in the isolated ED, consistent with the previous studies^25,27,28^. The difference in the formation energy between the stable and the metastable atom cores is not large at the GB, compared to the isolated ED. Thus, the variation of strain field near the GB is the major cause for presence of various atom cores, and the relatively small difference in formation energy is compensated by the strain energy.

**Table 1:** Formation energies of the atom cores observed at the Σ = 7 CSL boundaries and at the isolated EDs.

	5/7(S) core	5/7(M) core	8 core
Σ = 7 CSL boundary	5.33	6.13	5.71
ED	10.33	10.67	8.63

Energy values are given in eV per core.

**Figure 3:**
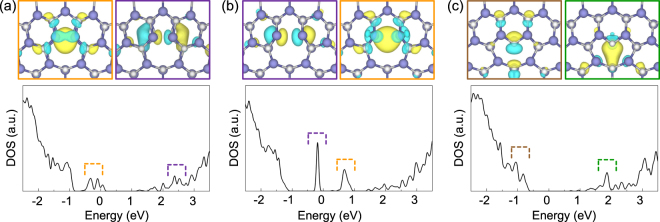
Wave functions corresponding to defect states localized at the cores, and the density of states (DOS) for the three types of cores: (**a**) 5/7(S), (**b**) 5/7(M), and (**c**) 8 atom cores. Each wave function was plotted from one defect state; the wave function and corresponding DOS are denoted by the colored box and dashed bracket.

Next, we investigated the defect states of each core structure by tracking the density of states (DOS) along with the wave functions corresponding to the defect states, as shown in Fig. 3 (and see Supplementary Fig. S2 for ED). Here, we determined the depth of the defect states based on the GaN bulk band gap, which was calculated to be 2.0 eV (Supplementary Fig. S1). Note that the Σ = 7 boundary and the isolated EDs have nearly identical defect states if they are composed of the same type of core structure. There is always a corresponding localized defect state between EDs and GBs. However, the energy levels of the isolated EDs are shifted slightly from those of the Σ = 7 boundary because two extended defects undergo different internal strains. Particularly in the GBs, both 5/7(S) and 5/7(M) atom cores have deep mid-gap states, but they exhibit different electronic structures. The 5/7(S) atom core exhibits only occupied states, whereas 5/7(M) had both occupied and unoccupied states in the band gap. The electronic states of 5/7(S) and 5/7(M) correspond respectively to the previous results calculated differently for 5/7 atom core^28,29^. On the other hand, the 8 atom core is inactive, because it has only shallow and resonant states. Therefore, the coexistence of various cores in high-angle GBs enables spatially inhomogeneous characteristics.

**Figure 4:**
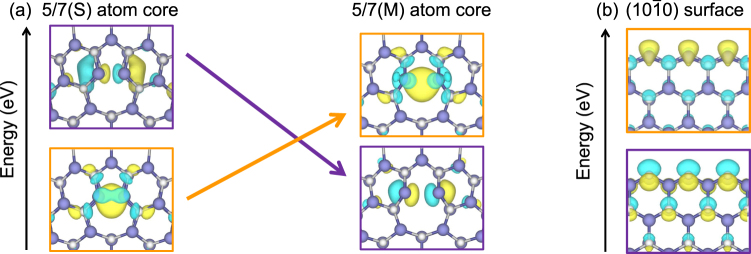
(**a**) Wave functions indicating the inversion of two localized states during the transition from 5/7(S) to 5/7(M). Wave functions enclosed by the orange and purple boxes represent the Ga 4*sσ* bonding state and N 2pσ* antibonding state, respectively. (**b**) Surface states in the (10$\overline{1}$0) surface, where the surface states with N 2*p* character were occupied.

It is noteworthy that defect states of 5/7(M) differ significantly from those of 5/7(S). In other words, reconstruction of the defect states occurs during the transition from 5/7(S) to 5/7(M). Importantly, the defect-state reconstruction is due to the inversion between two localized states, not due to the rehybridization of dangling bonds, as shown in Fig. 4(a); the occupied defect state of 5/7(S) becomes unoccupied in 5/7(M), and its unoccupied defect state becomes occupied in 5/7(M) during the transition from 5/7(S) to 5/7(M). In general, rearrangement among occupied states or among unoccupied states readily occurs under external strain. However, inversion between occupied and unoccupied states is an unusual phenomenon, for which there should be multiple mechanisms to stabilize the dangling bonds. We therefore further investigated the defect states in the 5/7 atom cores. The defect states denoted by the orange and purple dashed lines in Fig. 3(a,b) are characterized as the Ga 4*sσ* bonding and the N 2pσ* antibonding states, respectively, by looking at their corresponding wave functions shown in Fig. 4. Obviously, the Ga 4*sσ* bonding states become energetically favored by the bond formations in 5/7(S), while the N 2pσ* antibonding states remain unoccupied as shown in Fig. 4(a). During the transition from 5/7(S) to 5/7(M), the N 2pσ* antibonding (Ga 4*sσ* bonding) states become occupied (unoccupied) as the wrong bond of the 5/7 atom core is stretched. This phenomenon can be understood from the (10$\overline{1}$0) surface states with N 2*p* character, which are more stable than those with Ga 4*s* character, as shown in Fig. 4(b). Through this investigation, we found that the dangling bonds can be stabilized by bond formations or by surface relaxations in the 5/7 atom core, and competition between them is the source to induce the localized state inversion during the transition.

The high-resolution HAADF STEM analysis and *ab initio* DFT calculations showed the existence of the short-range order hidden in the high-angle GBs of GaN films. The type of relaxed core was correlated with the angle between two pairs of second-nearest bonds. These bonds can be observed in the nonperiodic boundaries, as well as in the periodic CSL boundaries. The latent order was identified by classifying the 5/7 atom cores into 5/7(S) and 5/7(M). The core relaxation order provides a physical understanding of the metastable cores (5/7(M) or 8 atom cores) existing in real GBs, depending on the surrounding strain. As a result, the degree of freedom at the high-angle GBs results in unique physical properties compared to those of the isolated EDs. We further analyzed the defect states of 5/7(S) and 5/7(M) and found that during the transition between two 5/7 atom cores, defect-state reconstruction was caused by localized state inversion rather than rehybridization of dangling bonds.

## Electronic supplementary material

Supplementary information
