# Supplementary material for: Latent Order in High-Angle Grain Boundary of GaN
Source: Sci Rep. 2018 Mar 15;8:4647. doi: 10.1038/s41598-018-22603-3 (PMC5854581; doi:10.1038/s41598-018-22603-3)
Supplement: Supplementary file 1 — Supplementary information [file 41598_2018_22603_MOESM1_ESM.pdf]

Supplementary Information

# Latent Order in High-Angle Grain Boundary of GaN

Sangmoon Yoon,[1,2] Hyobin Yoo,[1] Seoung-Hun Kang,[2,3] Miyoung Kim,[1,*] and Young-Kyun Kwon[2,3,†]

[1]*Department of Materials Science and Engineering, Seoul National University, Seoul, 08826, Korea*

[2]*Department of Physics and Research Institute for Basic Sciences, Kyung Hee University, Seoul, 02447, Korea*

[3]*Korea Institute for Advanced Study (KIAS), Seoul, 02455, Korea*

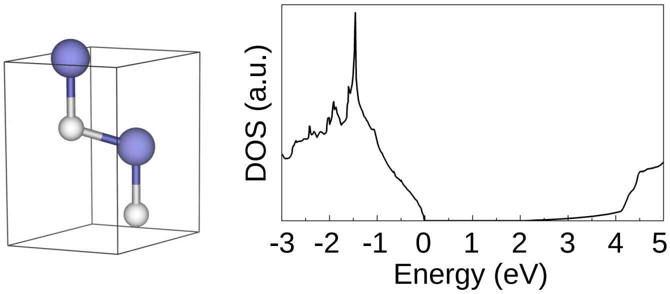


FIG. S1. Atomic structure and DOS of bulk GaN. The band gap of GaN was 2.0eV based on our calculation. The underestimation of band gap is the well-known issue of the LDA calculations.

* Corresponding author. E-mail: mkim@snu.ac.kr

† Corresponding author. E-mail: ykkwon@khu.ac.kr

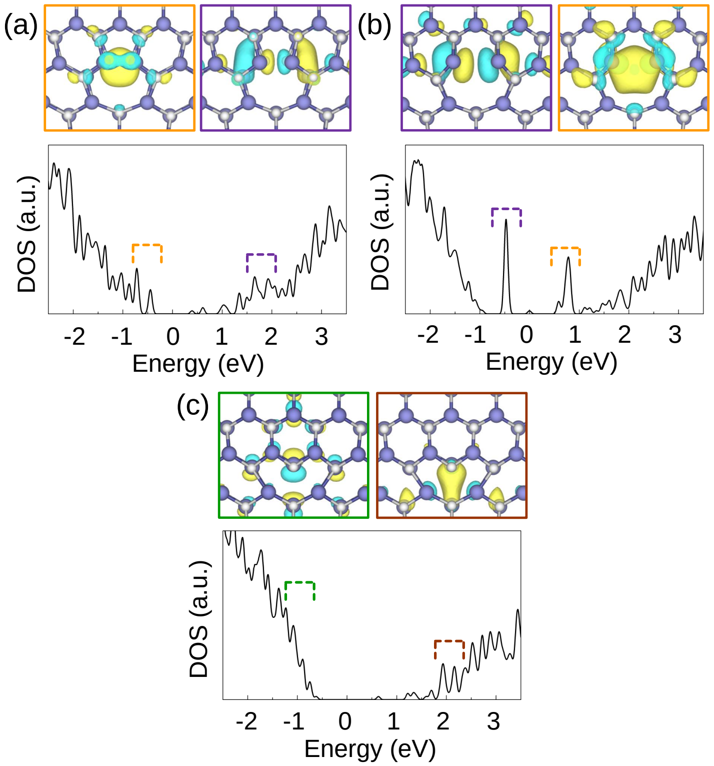


FIG. S2. Wave functions corresponding to defect states localized at the cores of edge dislocations (EDs) and DOS of them: (a) 5/7(S) atom core, (b) 5/7(M) atom core, and (c) 8 atom core. Each wave function enclosed by a specific-colored box was plotted from a defect state bracketed with the same-colored dashed line in the corresponding DOS graph. The localized states of EDs can be directly matched with those of $\Sigma = 7$ CSL boundaries in Fig. 3. However, the energy level of defect states are slightly shifted. For example, the mid-gap states of 5/7 atom cores are relatively shallow in the EDs than in the $\Sigma = 7$ CSL boundaries.
